# Development of Polyoxymethylene/Polylactide Blends for a Potentially Biodegradable Material: Crystallization Kinetics, Lifespan Prediction, and Enzymatic Degradation Behavior

**DOI:** 10.3390/polym11091516

**Published:** 2019-09-18

**Authors:** Jianhua Li, Yatao Wang, Xiaodong Wang, Dezhen Wu

**Affiliations:** 1 State Key Laboratory of Organic–Inorganic Composites, Beijing University of Chemical Technology, Beijing 100029, China; ecljh@kailuan.com.cn; 2 Coal Chemical R & D Center, Kailuan Group Limited Liability Corporation, Tangshan 063018, China; wangyatao@kailuan.com.cn

**Keywords:** polyoxymethylene, polylactide, blends, crystallization kinetics, potential biodegradation, lifespan prediction

## Abstract

This paper reported the development of polyoxymethylene (POM)/polylactide (PLA) blends for a potentially biodegradable material. A series of POM/PLA blends at different weight ratios were prepared by melt extrusion with a twin-screw extruder, and their mechanical properties, crystallization behavior and kinetics, thermal degradation kinetics and stability, lifespan prediction and enzymatic degradation behavior were investigated extensively. POM and PLA were found to be partially miscible in the melt state at low temperature and become phase-separated at elevated temperatures, and their blends exhibited a typical lower critical solution temperature behavior. There were two distinct glass transition temperatures (*T_g_*) observed for POM/PLA blends at any mass ratios when quenched from the homogeneous state, and both POM and PLA domains showed an apparent depression in their respective *T_g_*’s in the blends. Owing to the partial miscibility between two domains, the tensile strength and impact toughness of POM/PLA blends gradually decreased with an increase of PLA content, but their flexural strength and modulus presented an increasing trend with PLA content. The studies on non-isothermal and isothermal crystallization behaviors of the blends indicated that the crystallization rates of the blends decreased continually with increasing the PLA content, confirming that the crystallization of POM domain was controlled by the molecular-confined mechanism. The introduction of PLA into POM not only led to a slight increase of thermal stability of POM domain at low PLA contents but also shortened the lifespan of the blends, favoring the natural degradation of the blends. The POM/PLA blends exhibited an improvement in partially biodegradable performance with an increase of PLA content and their mass loss reached up to 25.3 wt % at the end of 48-h enzymatic degradation when 50 wt % of PLA was incorporated.

## 1. Introduction

As sustainable development has become a worldwide tendency, there is growing awareness of the importance of biodegradable polymeric materials and their products in our ordinary daily life. Nowadays, human beings have to face a series of global challenges including greenhouse gas emissions, global warming, climate change fossil energy shortage, and environmental pollution [1]. It has been well known that most of polymeric materials are produced from petroleum feed stocks/coal and eventually end up as non-degradable waste at the end of their service life. These pollutants from polymeric materials inevitably influence the soil, rivers, lakes, oceans and even food chain on which human beings depend. Moreover, the disposal of polymer wastes by incineration also leads to enormous environment pollution while reducing landfill sites. These factors have contributed to the development of environmentally friendly polymers that degrade completely under composting conditions after the end of their service life. Therefore, environmental pollution resulting from the careless disposal of bioinert polymeric materials is becoming a serious and critical problem all over the world. The exploitation of renewable and biodegradable materials and the restrictions of the use of traditional polymeric materials are considered as two important means to avoid an overdependence on fossil energy resources, which can effectively reduce the environmental pollution and carbon emissions [2].

Polylactide (PLA) is a biodegradable semi-crystalline polymer that is manufactured by biotechnological processes from renewable resources such as corn or sugarcane [3], and it is considered as a promising sustainable material because of its biomass-origin, recyclability, and biodegradability [4]. PLA has attracted great interest in recent years and been widely used for biomedical applications because of its reasonable mechanical properties and good biocompatibility [5,6] and became an alternative to traditional commodity plastics for everyday applications as an environmental friendly polymer due to its unique properties such as high strength, high stiffness, resistance to fats and oil [7,8,9]. However, PLA has a much higher cost compared to many of petroleum-based thermoplastics for disposable applications. Furthermore, the limited mechanical properties and complicated processing procedures also hinder its practical applications in a wider range [10]. To overcome this limitation, partially biodegradable polymers have been developed as a compromise between cost and performance. Most of the polymers may achieve desirable degradation through two major pathways: The designs of a polymer from monomers, which are prone to microorganisms, and the incorporation of biodegradable additives or groups into the polymer. This in turn can be conducted by two methods. The first method involves the copolymerization of biodegradable monomers with the non-degradable monomer, and the second one involves the blending of a biodegradable additive/polymer with a non-degradable polymer [11]. It has been widely accepted that polymer blending is one of the most important ways to develop new high-performance polymeric materials [12]. Polymer blends with different physical properties often exhibit the possibility of enhancing the overall properties of a material through a synergistic combination of desirable properties from the original polymers. Gajria et al. [13] investigated a blending system based on PLA and poly(vinyl acetate) prepared by a single-screw extruder and found that the resulting blends containing 5 and 10 wt % of PLA presented higher tensile strength than pure PLA as well as good biodegradability. Kim et al. [14] studied the mechanical, morphological, and rheological properties of polycarbonate/PLA blends with various compatibilizers and found that the blends exhibited modest hydrolytic degradation with the addition of poly(styrene-g-acrylonitrile) grafted with maleic anhydride as the most effective compatibilizer. Lehman et al. [15] designed a biodegradable polymeric system based on PLA and poly(methyl methacrylate) via melt processing and observed a fine interconnected structure in this blending system, which appears attractive for fabricating certain biomaterials. McCarthy et al. [16] reported an investigation on the enzymatic degradation behavior of PLA/poly(ethylene glycol) blends and found that the weight loss for all of the blends was significantly greater than that of pure PLA due to the dissolution of PEG and the degradation of PLA. Y. Agari et al. [17] also prepared a series of PLA/poly(ethylene oxide) by the dissolution-diffusion process and observed that all the blends were degraded more rapidly than pure PLA due to the dissolution of PEO with water, which increased the surface area attacked by the enzyme. Moreover, the other biodegradable blending systems such as polypropylene/PLA [18], polyethylene/PLA [11], polystyrene/PLA [19], and thermoplastic polyurethane/PLA [20] blends were also studied intensively for use in biomaterials. These studies clearly demonstrated that the specific applications of biodegradable blends had received significant attention in offering an attractive route to further improve environmental waste management [11,21,22,23,24].

Polyoxymethylene (POM) is an important engineering thermoplastic that was derived from formaldehyde synthesis, and it has a good balance in mechanical performance, excellent creep and fatigue resistance, good electrical properties, low friction coefficient and excellent anti-wear properties [25]. Although POM can well meet the requirement for highly demanded mechanical, electronic, constructional, and automotive applications, it is not biodegradable due to its stable molecular structure [26,27,28]. The most of waste POM have only to be treated by burial or incineration, which is easy to cause secondary pollution. It is desirable that partially biodegradable performance can be achieved by blending POM with a biodegradable polymer like PLA. According to a literature survey, the POM/PLA blends have been determined as a partially miscible blending system in the melt state at low temperatures by Qiu et al. [29] and Ye et al. [30]. Qiu et al. [31] also found that the crystallization rate of PLA domains could be enhanced by POM fragment crystals in the PLA/POM blends with a small amount of POM. Furthermore, Zhu et al. [32] reported a parallel-stripe structure observed in PLA/POM blend films, and Ye et al. [33] reported a crystallization-modulated nanoporous structure for PLA/POM blends with hierarchical patterned surfaces and 3D interpenetrated internal channels due to such a partially miscible behavior. Huan et al. [34] reported an investigation on the morphology, crystallization, rheology, and mechanical properties of PLA/POM blends so as to improve the heat resistance of PLA. Moreover, Mathurosemontri et al. [35] studied the effect of injection speed on morphology and mechanical properties of POM/PLA blends and found that high injection speed could enhance phase distribution of PLA in the POM domain and thus improved the mechanical properties of the blends. However, these studies mainly focused on the fundamental issues of polymer physics in the POM/PLA blending system, and they are seldom involved in the application development. A systematical and rigorous investigation on the crystallization, thermal degradation, and enzymatic degradation behaviors of POM/PLA blends have not been reported by now. Considering the combination of POM with PLA as a biodegradable polymer, the possible biodegradability may be achieved for the resulting blends. In this work, we attempted to develop a potentially biodegradable material based on POM and PLA through a melt blending method. As the miscibility of POM with PLA was confirmed, the mechanical performance and morphology of POM/PLA blends were evaluated. The crystallization behaviors and relevant kinetics of the blends were studied under the isothermal and non-isothermal conditions, their thermal degradation behaviors, corresponding kinetics and predicted lifetime were further investigated intensively, and the degradation behaviors were revealed under enzymes. The aim of this study is to exploit a novel type of potentially biodegradable material based on the POM/PLA blends for sustainable applications.

## 2. Experimental Section

### 2.1. Materials

Polyoxymethylene (POM) resin used in this work was kindly provided by Kailuan Group Co., Ltd., Tangshan, China, and it has a number-average molecular weight of around 30,000 and a melt flow index of 13.0 g/10 min. A commercial grade product of PLA resin (3052D) with a melt flow index of 14.0 g/10 min was purchased from Nature Works Co. LLC, Minnetonka, MN, USA. This PLA resin has a number average molecular weight of 72,300 g/mol and an l-lactide content of 96.5 wt %. The antioxidants (IRGANOX^®^ 245 and IRGAFOS^®^ 168) were purchased from BASF East Asia Regional Co., Ltd., Shanghai, China. Phosphate buffer solution (PBS) (pH = 7.2) and the solution containing 20,000 unit/mL of penicillin-streptomycin (PS) were purchased from Sigma-Aldrich China-Mainland Co., Ltd., Shanghai, China.

### 2.2. Preparation Methods

The blends of POM and PLA at different weight ratios were prepared by a facile melt-blending method. In a typical processing procedure, PLA and POM resins were dried in a vacuum oven at 80 °C for 24 h prior to use. POM, PLA, and various processing additives were premixed in a high-speeding mixer and then blended through melt extrusion using a ZSK Mc^18^ co-rotating twin-screw extruder (Coperion (Nanjing) Machinery Co., Ltd., Nanjing, China) with a screw diameter of 32 mm and an L/D ratio of 40:1. The rotation speed of the screw was set at 120 rpm, and the temperatures were set to 165, 175, 180, 185, 190, 185 and 180 °C from feeding zone to extruding die. A reasonable throughput of 30–35 kg/h was maintained for the blends during melt extrusion under this processing condition. The extruded strands were cooled in a water bath and then cut into pellets using a pelletizer. The obtained compounds were dried in a vacuum oven at 80 °C for 12 h to minimize the effects of moisture and then were injection molded into the standard test bars with different shapes for further characterizations and measurements using a Haitian injection molding machine under an injection temperature of 185 °C, an injection pressure of 60 MPa, and an injection speed of 60 mm/s.

### 2.3. Characterizations and Measurements

Differential scanning calorimetry (DSC) measurements were performed to characterize the glass transition behaviors of POM/PLA blends using a DSC 8000 differential scanning calorimeter (PerkinElmer Instruments, Richmond, CA, USA) equipped with a Universal Analysis 2000 data station at a heating rate of 10 °C/min in nitrogen atmosphere. The isothermal and non-isothermal crystallization behaviors of the blends were also investigated with the same DSC instrument. To record non-isothermal crystallization exothermic thermograms, the specimens were heated up to 190 °C at a rate of 20 °C/min and then held at this temperature for 5 min in order to erase their thermal histories. Afterward, the specimens were cooled from 190 to 30 °C at four given scanning rates of 2.5, 5, 10, and 20 °C/min to perform non-isothermal crystallization. The recorded non-isothermal crystallization exothermic thermograms were analyzed using the Jeziorny-modified Avrami model [36]. As for the evaluation of isothermal crystallization behaviors, the specimens were heated to 190 °C at a scanning rate of 20 °C/min and rapidly cooled to four given crystallization temperatures of 146, 148, 150, and 152 °C at a cooling rate of 50 °C/min, and then kept at the same temperature to perform isothermal crystallization until the crystallization process was complete. The recorded isothermal crystallization exotherms were analyzed using the Avrami model [37,38].

Polarized optical microscopy was performed to observe spherical morphology of POM/PLA blends on an Olympus BX51 polarizing microscope (Olympus Corporation, Tokyo, Japan) equipped with a Linkam THMS 600 temperature controller and a Sony CCD-IRIS digital camera. The specimens were heated to 190 °C on a hot plate, held at this temperature for 3 min, and then cooled to a given crystallization temperature of 145 °C, at which the growing of spherulites started. The samples were held at this temperature for 2 h to observe the change in spherulite size. The cloud temperatures of the blends were also detected by the same polarizing microscope. A thin film was first prepared by hot compression for the blend and then observed continuously by the microscope on a thermal platform with a heating rate of 1 °C/min. The temperature was recorded as the cloud point when a transition from transparence to cloud was distinguished. Tensile and flexural tests were carried out using an Instron 5966 universal tensile testing machine (Instron Coporation, Boston, MA, USA) according to the standard ISO 527 and ISO 178 test methods, respectively. The tensile tests were performed at a crosshead speed of 50 mm/min, and the flexural tests were performed at a crosshead speed of 2 mm/min and a support span of 64 mm. Izod impact tests were conducted on an CEAST 9010 impact test machine (Instron Corporation, Boston, MA, USA) according to the standard ISO 180 test method at a pendulum energy of 2.75 J. All of the mechanical measurements were carried out at a room temperature around 25 °C and 50% relative humidity. At least five specimens were tested for each sample and the reported mechanical data reflected an average from five tests. 

Dynamic mechanical analysis (DMA) was carried out with a Q800 apparatus (TA Instruments, New Castle, DE, USA) using the standard test bar with a dimension of 54 mm × 10 mm × 4 mm. All the measurements were performed in the linear region with the strain of 0.01%. Storage moduli, loss moduli, and loss factors (tan δ) were determined at a frequency of 1 Hz and a scanning rate of 2 °C/min as a function of temperature from −100 to 150 °C. Scanning electron microscopy (SEM) was performed to observe the surface morphologies of the impact fracture of POM/PLA blends on an S–7800 scanning electron microscope (Hitachi High-Technologies Corporation, Tokyo, Japan). Fourier-transform infrared (FTIR) spectra of the blends were recorded on a Nicolet Nexus 670 infrared spectrophotometer (Thermo Fisher Scientific (China) Co., Ltd., Shanghai, China) in the wavenumber range of 4500–400 cm^−1^ with a scanning number of 32 and a resolution of 4 cm^−1^. The test specimen was sandwiched between a pair of KBr plates for FTIR measurement. Raman spectra were obtained on a B&W TEK InnoRaman-785H spectrometer (Metrohm AG, Herisau, Switzerland) with the CleanLaze™ argon ion laser as an excitation source using the 785 nm line. X-ray powder diffraction (XRD) was performed on a D2 X-ray diffractometer (Bruker Nano GmbH, Berlin, Germany) using Cu-Kα radiation (λ = 0.154 nm) and operated at 2θ from 5° to 50° with a scanning rate of 2°/min under 40 kV and 40 mA.

Thermogravimetric analysis (TGA) was carried out to analyze the thermal decomposition behaviors of POM/PLA blends on a TGA 4000 thermogravimetric analyzer (PerkinElmer Instruments, Richmond, CA, USA) under a dynamic N_2_ gas atmosphere at a flow rate of 20 mL/min. TGA experiments were conducted at four given scanning rates of 5, 10, 15, and 20 °C/min over a range of temperature from 50 to 600 °C. The specimen mass was kept for 2.5 ± 0.2 mg and *α*-Al_2_O_3_ was used as a reference material for all of the TGA measurements. The thermal degradation kinetics of the blends was analyzed using the Flynn and Wall’s method [39], and their lifetime were estimated according the method proposed by Toop et al. [40].

The enzymatic degradation performance was measured according to the method reported by Wang et al. [41,42] The specimen sheets obtained from POM/PLA blends were placed in 100 mL of phosphate buffer solution containing 6.0 mL of PS solution and then incubated at 37 °C for 40 days. The specimens were taken at a certain interval. The removed resins were washed repeatedly with deionized water and accurately weighed after vacuum drying. The mass loss was calculated, and the surface morphologies were observed with SEM.

## 3. Results and Discussion

### 3.1. Miscibility Analysis

**Figure 1:**
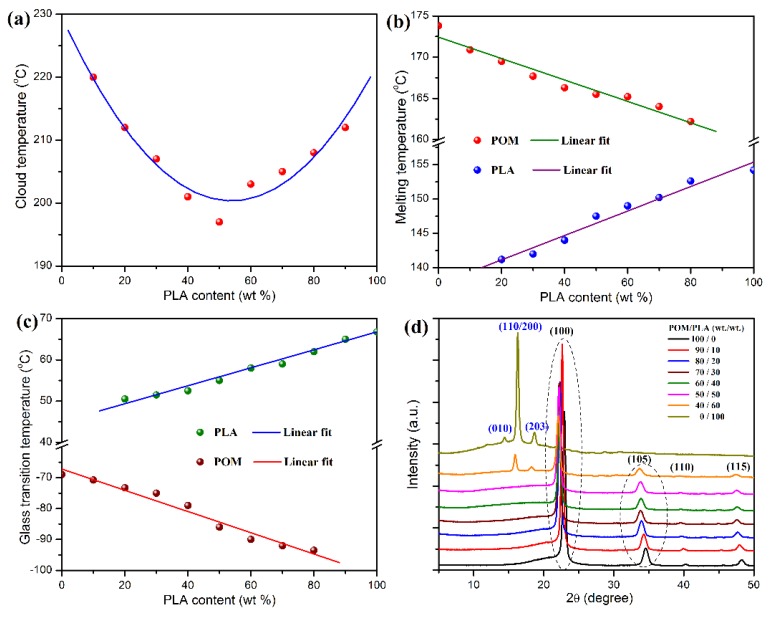
(**a**) Cloud temperatures, (**b**) melting temperatures, (**c**) glass transition temperatures, and (**d**) XRD patterns of polyoxymethylene/polylactide (POM/PLA) blends at different mass ratios.

It is well known that a miscible polymer blend usually has a single-phase structure and presents the average properties depending on the compositions of this blend [43,44]. Oppositely, the immiscibility between two polymers can lead to a series phase separation and inevitably deteriorates the mechanical performance of their blending system. Therefore, it is vital to seek a miscible system for polymer blends so as to gain desirable properties. Although it has long been known that POM is seldom miscible thermodynamically with other polymers because of its specific molecular structure and crystallinity, it still indicates partially miscibility with PLA on the basis of some publications. In this work, we intended to reconfirm this thermodynamical miscibility by means of a series of typical characterization results in Figure 1. As seen in the phase diagram of POM/PLA blends in Figure 1a, this blending system exhibits a series of typical cloud temperatures dependent on the blending compositions due to a phase separation occurring at the critical solution temperature. Moreover, a lower critical solution temperature (LCST) was observed at around 185 °C for the blend with a POM/PLA mass ratio of 50/50, suggesting a typical miscible behavior of POM/PLA blends [29]. The incorporation of PLA also leads to a decreasing trend in melting temperature (*T_m_*) of POM as a function of the PLA content as shown in Figure 1b, in which a similar descent trend could be observed for the *T_m_* of PLA as a function of the POM content. It is understandable that the miscible PLA chains may disturb the crystallization process of POM chains due to the interaction between two domains in the melt state, resulting in an imperfect crystalline form accordingly. In this case, the reduction of *T_m_* for a semi-crystalline POM domain is considered as an indicator for its miscibility with PLA. In addition, it is noteworthy that the LCST of POM/PLA blends is far higher than its *T_m_*, indicating that the blends always keep a homogeneous state due to their miscible domains at low temperature even in the melt state but have to suffer a phase separation at the temperature much higher than *T_m_*.

On the other hand, the variation trend in glass transition temperature (*T_g_*) can be taken as another indicator for a miscible polymer blending system, and only one *T_g_* is a vital criterion for the miscibility of polymeric components. However, there are two dependent *T_g_*’s observed in the POM/PLA blending system according to the DSC results shown in Figure 1c. Pure POM and PLA exhibit the *T_g_*’s of −73 and 66 °C, respectively. It is noteworthy that the *T_g_* of POM domain presents a decreasing trend as a function of the PLA content, while the PLA domain shows an opposite trend for its *T_g_*. Such a shift of *T_g_* with a variation of weight fraction of each domain is ascribed to the occurrence of phase separation between two domains due to partial miscibility, which increases the free volume for both macromolecular chains and results in a reduction of *T_g_* accordingly [29]. XRD measurement was performed to confirm the effect of miscibility on the crystallinity of POM and PLA, and the resulting XRD patterns are given in Figure 1d. Although the XRD patterns of POM/PLA blends clearly show the diffraction peaks of each domain, there is a slight fluctuation in the 2θ value observed on the (100) and (105) reflections of the POM domain with a variation of PLA content. This phenomenon can be explained by the fact that the macromolecular entanglement between POM and PLA may disturb the crystallization of POM domain, thus leading to the formation of imperfect crystals of POM domain. In this case, the Bragg angle tends to fluctuate with a variation of the PLA content.

**Figure 2:**
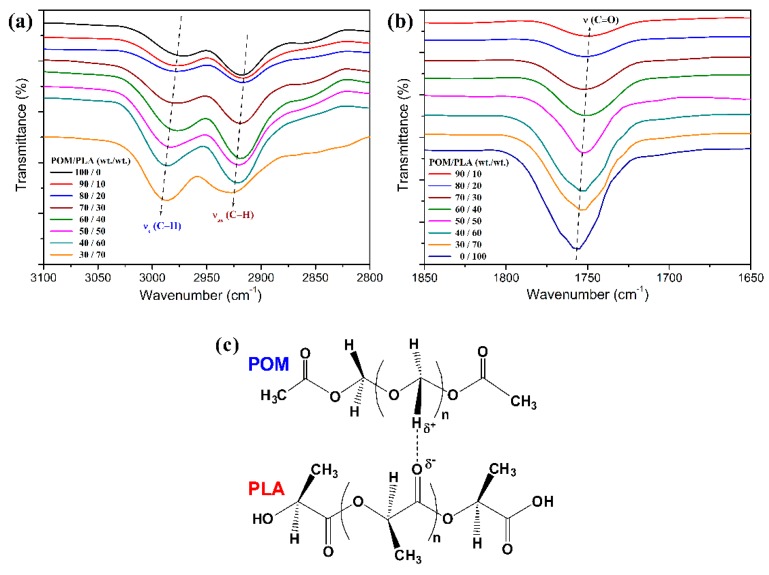
(**a**,**b**) FTIR spectra of POM/PLA blends at different mass ratios; (**c**) schematic interaction between the POM and PLA molecules.

To further confirm the driving power of miscibility between POM and PLA, FTIR spectroscopy was performed to detect the variation of characteristic absorption bands of POM and PLA domains. Figure 2 shows the infrared spectra of POM/PLA blends at different mass ratios, which may reflect the potential interaction between two domains. As seen in Figure 2a, pure POM exhibits two characteristic absorption peaks at 2975 and 2918 cm^−1^ to the symmetric and asymmetric stretching vibrations of C−H bond, respectively. It is interesting to note in the infrared spectra of the blends that these two characteristic bands reveal a slight and constant shift to higher wavenumber with an increase of PLA loading. On the other hand, pure PLA shows a characteristics peak at 1756 cm^−1^ due to the stretching vibration of its carbonyl groups as observed in Figure 2b. This characteristic absorption band in the infrared spectra of the blends is found to continually shift to lower wavenumber with an increase of PLA loading. Qiu et al. [29] observed the similar interesting band shifts occurring in POM and PLA domains from their blends and they considered the possible formation of hydrogen bonding between the POM and PLA molecular chains as schematically illustrated in Figure 2c. They thought that the intrachain dipolar interaction of POM domains was reduced because of the hydrogen bond of C−H····O=C, thus resulting in a shift to higher wavenumber for the corresponding absorption bands as supported by Lee et al. [45]. However, considering the fact that the vibrational frequencies of both domains in the partially miscible blends are influenced to a much greater extent than the reported explanations, such band shifts are more likely ascribed to the phase-separated structures in the blends.

### 3.2. Mechanical Properties and Fracture Morphology

**Figure 3:**
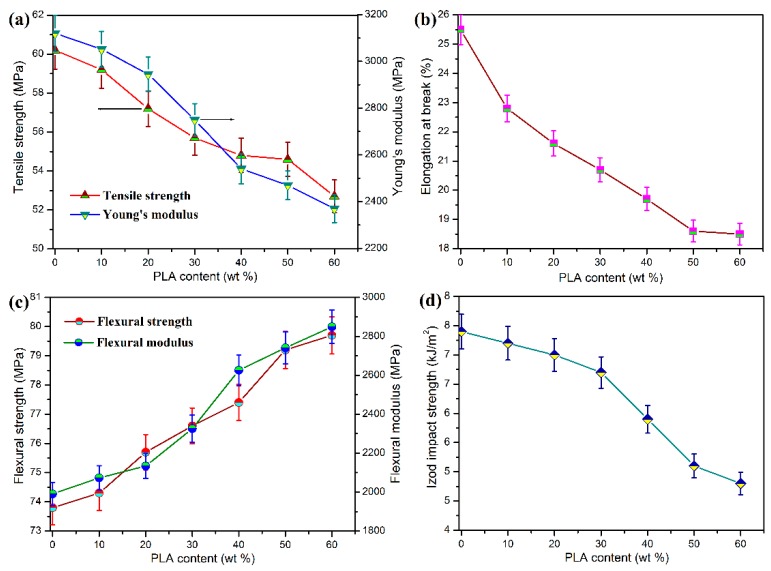
(**a**) Tensile strength and Young’s moduli, (**b**) elongation at break, (**c**) flexural strength and moduli, and (**d**) Izod impact strength of pure POM and its blends with PLA.

**Figure 4:**
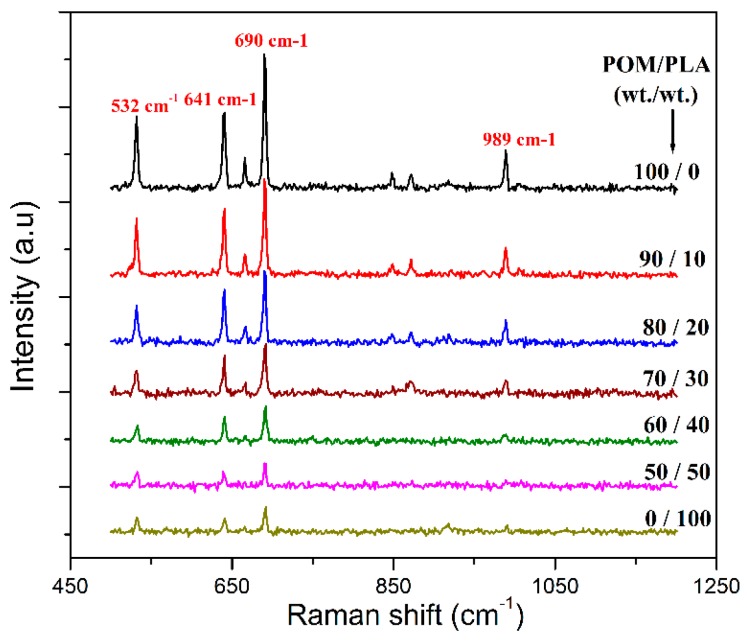
Raman spectra of pure POM, PLA and their blends.

The mechanical performance of POM/PLA blends was evaluated in terms of tensile, flexural, and notched impact measurements, and the obtained data are presented in Figure 3. Pure POM is found to show pretty high tensile and flexural strength and moduli as well as good impact toughness according to the mechanical data in Figure 3. It has been broadly reported that POM is miscible with few of polymers due to its special chain structure, and only thermoplastic polyurethane (TPU) has been generally recognized as an impact modifier for POM by now [46]. Nevertheless, the combination of POM and TPU brings about a serious decrease in tensile and flexural properties when gaining a good toughening effect. It is found that the blending system at the POM/PLA mass ratios of 90/10 and 80/20 exhibits a slight reduction in tensile strength and modulus as well as Izod impact strength but an improvement in flexural strength and modulus. The interface existing between the POM and PLA phases may weaken the stress transfer during the tensile and impact fracture in spite of the partial miscibility of POM with PLA. The decrement in tensile and impact performance is still limited compared to other immiscible POM-based blending systems [29,35]. However, there are highly negative results in tensile and impact performance when the PLA loading is higher than 30 wt %, implicating that the stress transfer effect is deteriorated significantly with an increase of additional phase. It has been reported that PLA has inherent brittleness and a high sensitivity to notched impact, which are due to its low entanglement density and high value of characteristics ratios [18]. The decrease of impact toughness of POM/PLA blends may be due to the additive behavior resulting from the increase of interface between two phases [35], which is related to the distribution of POM and PLA domains from the surface to the core of mechanical test bar of the blends. To understand the effect of domain distribution in the test bar, Raman spectroscopy was conducted to analyze the molecular structure and composition of material surfaces, and the resulting spectra are displayed in Figure 4. It is observed that both pure POM and PLA exhibit a set of Raman peaks at 532, 641, and 690 cm^−1^ assigned to their backbone chemical structure. Pure POM is found to show a higher Raman shifting intensity than pure PLA at the peak of 532 cm^−1^ due to its hexagonal structure. Moreover, pure POM also presents a characteristic Raman shift at 989 cm^−1^ associated with its unique monotonic structure. It is observed in Figure 4 that the four Raman characteristic peaks also appear in the Raman spectra of POM/PLA blends, but their shift intensity declines with an increase of PLA loading. Furthermore, there is a significant enhancement in shift intensity of these Raman peaks, especially at 989 cm^−1^ when the PLA loading is lower than 30 wt %. This phenomenon suggests that the sample surface of the blends is mainly composed of a POM-rich domain when the PLA loading is lower than 30 wt %, and however there is a transition from the POM-rich domain to the PLA-rich one on the sample surface with the PLA loading over 30 wt %. Such a transition enlarges the domain size of PLA and leads to an increase in interphase tension between the POM and PLA domains accordingly. This result is disadvantageous for the mechanical strength of POM/PLA blends. As a result, the deterioration of tensile and impact strength becomes more significant.

**Figure 5:**
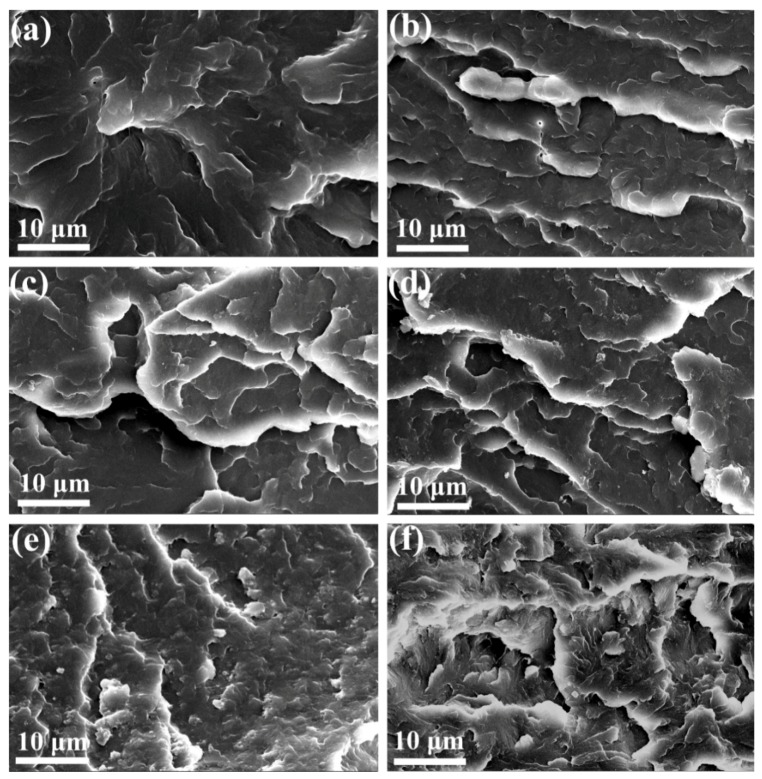
SEM micrographs of impact fracture surfaces of (**a**) pure POM and its blends with (**b**) 10 wt %, (**c**) 20 wt %, (**d**) 30 wt %, (**e**) 40 wt %, and (**f**) 50 wt % of PLA.

The morphologies of impact fracture surfaces of POM/PLA blends were further investigated by SEM, and the obtained micrographs are illustrated in Figure 5. As noted in Figure 5a, pure POM reveals a smooth fracture surface with small matrix deformation, indicating a typical morphology from brittle fracture. On the other hand, the blends also exhibit a uniform surface with increasingly enhanced deformation in the PLA loading range of 10–30 wt %, and there is no separated phase or clear boundary between two phases observed as seen in Figure 5b–d. When the PLA loading is improved to 40 and 50 wt %, such a homogeneous phase structure is still maintained, followed by more serious matrix deformation. Although the interface is still hard to be distinguished between the POM and PLA phases, some wrapped spherical particles seem to appear on the fracture surface as the dispersed phase of PLA (see Figure 5e,f). These SEM observation results confirm a homogeneous phase structure in the blends even at a high loading of PLA due to the miscibility between two polymers, and therefore the deterioration in mechanical performance of the blends is effectively depressed. In summary, it is still a valuable route to gain the biodegradability for POM/PLA blends at a low loading of PLA even if there is a limited loss in tensile and impact performance compared to non-biodegradable pure POM resin.

### 3.3. Dynamic Mechanical Analysis (DMA)

**Figure 6:**
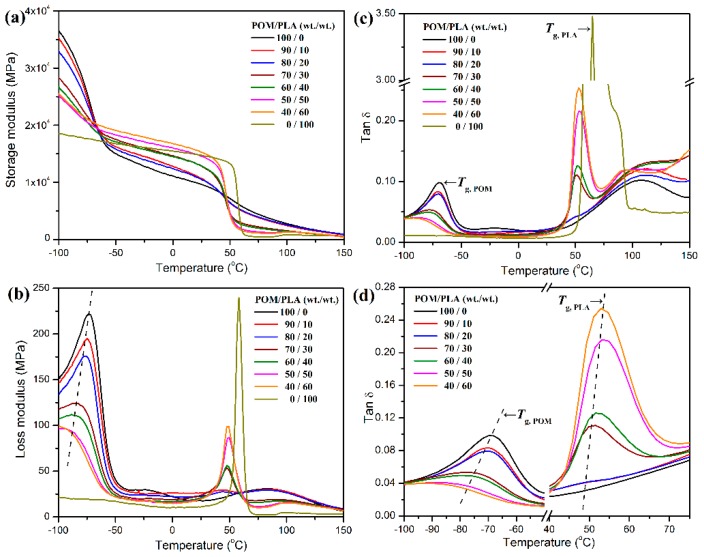
(**a**) Storage moduli, (**b**) loss moduli, and (**c**,**d**) loss factors (tan δ) of pure POM, pure PLA, and their blends as a function of temperature.

The dynamic mechanical properties of POM/PLA blends were investigated by DMA. Figure 6 shows the plots of storage moduli, loss moduli, and loss factors (tan δ) as a function of temperature for pure POM, pure PLA, and their blends. Pure POM is found to present at a much higher storage modulus than PLA before the glass transition occurs, indicating a better load-bearing capacity at low temperature because of a high rigid nature of POM. There is a sharp drop in storage modulus observed at around −48 °C related to the glass transition of POM. The incorporation of PLA into POM leads to a decline in storage modulus due to the presence of a soft PLA domain in the POM matrix, and such a decline seems to be more notable with an increase of PLA content. However, an opposite trend is observed for storage modulus at the temperature higher than the *T_g_* of POM, which is attributed to the activated motion of the chain segments of POM macromolecules. This trend is in good agreement with that of the flexural modulus measured at room temperature. It is also observed in Figure 6b that both of the pure resins show a sharp relaxation peak corresponding to glass transition in their plots of loss modulus. However, all of the POM/PLA blends exhibited two separated loss modulus peaks for individual components as a result of the presence of a non-homogeneous phase in the blends. This confirms the formation of a dual-phase structure in the blends. Moreover, it is noteworthy that the addition of PLA not only results in a gradual reduction of loss modulus at relaxation peak temperature, but also leads to a shift of relaxation peak. This phenomenon is ascribed to the presence of phase boundary, and moreover, the introduction of PLA phase may hinder the molecular motion of POM phase in amorphous regions. As a result, macromolecular relaxation was depressed, and the relevant loss modulus was reduced. The similar trends can be found from the plots of loss factor, in which there are also two independent loss factor peaks distinguished clearly. For the POM/PLA blends at a mass ratio of 90/10, the lower temperatures peak of loss factor at around −70.2 °C is attributed to the *T_g_* of POM domain and the higher one at about 49.3 °C corresponds to the *T_g_* of PLA domain. As observed in Figure 6c,d, the loss factor peak of POM domain shifts to a lower temperature with an increase of PLA content, and meanwhile the loss factor peak of PLA domain shifts to a higher temperature. These results are consistent with the findings in DSC investigation and confirm that the POM domain is partially miscible with the PLA one in the blend system.

### 3.4. Crystallization Behaviors and Kinetics

**Figure 7:**
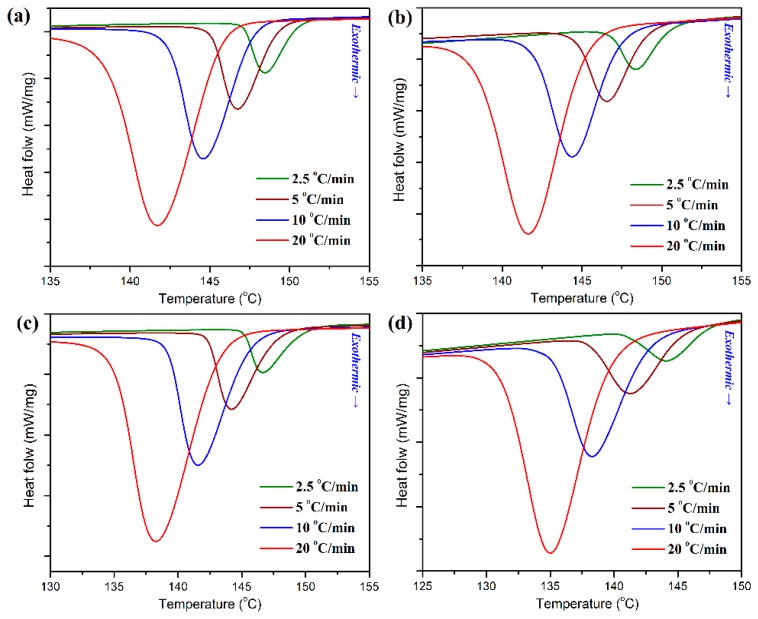
Differential scanning calorimetry (DSC) thermograms for the non-isothermal crystallization of (**a**) pure POM and its blends with (**b**) 10 wt %, (**c**) 20 wt %, and (**d**) 40 wt % of PLA at different cooling rates.

POM is a typical semi-crystalline polymer with a high degree of crystallinity, and it usually forms a complex heterogeneous system composed of amorphous and crystalline phases of a different order and hierarchical crystalline structure during the crystallization process. Therefore, the crystallization behavior of POM plays an important role in its production processing such as injection molding, extrusion molding and blow molding, and directly affects the properties of molded products. For example, the mechanical performance of POM is strongly associated with the molding process, especially pronounced in injection-molded devices [35]. On the other hand, the introduction of other polymers also influences the crystallinity and crystallization behavior of POM in most cases. In this work, considering that the industrial processing conditions strongly depend on its crystallization performance with technological importance in the processability of POM/PLA blends, it is essential to well understand the crystallization behaviors and kinetics of POM domain in its blends with PLA. The non-isothermal crystallization behaviors and kinetics of POM/PLA blends were first studied by using DSC at different cooling rates, and the obtained DSC curves are illustrated in Figure 7. As observed in Figure 7, pure POM display a single crystallization peak (*T_p_*) at 148.5 °C in its DSC thermogram with a cooling rate of 2.5 °C/min, and however the *T_p_* is found to shift to a lower temperature with an increase of cooling rate, followed by a significant enhancement in the peak intensity of heat flow. It is understandable that a higher cooling rate corresponds to a smaller timescale for crystallization, and therefore a higher supercooling degree is required to initiate the crystallization of POM, thus leading to a longer crystallization time for POM. Although the POM/PLA blends show a similar trend in the variation of *T_p_* for POM domain, the *T_p_*’s of the blends seem to be lower than that of pure POM and tend to decrease with an increase of PLA content at all of the cooling rates. Considering the fact that the PLA domain has much lower crystallization rate and temperature than the POM one, the molten PLA evidently disturbs the crystallization of POM domain and results in a decline of *T_p_*.

**Figure 8:**
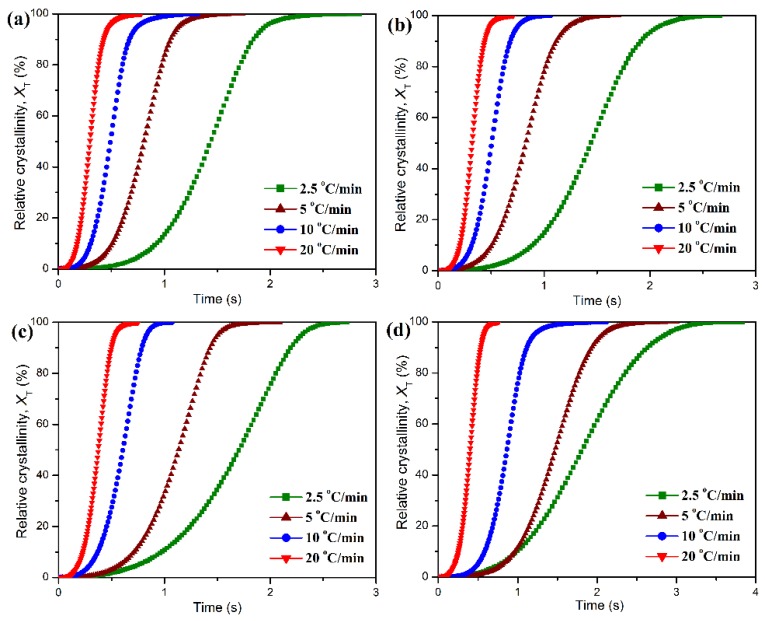
Development of relative degree of crystallinity as a function of time for the non-isothermal crystallization of (**a**) pure POM and its blends with (**b**) 10 wt %, (**c**) 20 wt %, and (**d**) 40 wt % of PLA at different cooling rates.

The development of relative degree of crystallinity as a function of temperature can be derived from these DSC diagrams by Equation (1) under the non-isothermal condition, and the resulting plots are presented in Figure 8.

(1) $$X_{T}=\frac{\int_{T_{0}}^{T}\left(\frac{dH_{c}}{dT}\right)dT}{\int_{T_{0}}^{T_{\infty }}\left(\frac{dH_{c}}{dT}\right)dT}\times 100\%$$

where *X_T_* is the relative degree of crystallinity, *dH*_c_/*dT* the heat flow of DSC scan, *T* an arbitrary temperature, and *T*_0_ and *T*_∞_ are the crystallization onset and end temperatures, respectively. All of the development plots of *X_T_* exhibit a sigmoid mode, but the crystallization period of POM domain seems to be prolonged with the addition of PLA. The non-isothermal crystallization time (*t*) can be deduced from Equation (2).

(2) $$t=\frac{T_{0}-T}{\phi }\times 100\%$$

where *ϕ* is the cooling rate, *T*_0_ the onset crystallization temperature at *t* = 0, and *T* is the temperature at crystallization time *t*. To obtain the kinetic parameters, the experimental data based on the plots of *X_T_* as a function of *T* should be converted to the relative degree of crystallinity (*X_t_*) as a function of time by Equation (2). In this case, a crystallization kinetic analysis was conducted to evaluate the crystallization behaviors of POM and its blends with PLA using the Jeziorny-modified Avrami model with an expression as follows [36]:

(3) $$1-X_{t}=\exp(-Zt^{n})$$

where *X_t_* is the relative degree of crystallinity at time *t*, *n* the Avrami exponent that depends on the nucleation and growth mechanism and *Z* is the growth rate constant involving both nucleation and growth rate parameters. The values *n* and *Z* are determined from the slope and intercept of the plot of ln[−ln(1 − *X_t_*)] versus ln*t* in terms of Equation (4).

(4) $$\ln\left[-\ln\left(1-X_{t}\right)\right]=n\ln t+\ln Z$$

**Figure 9:**
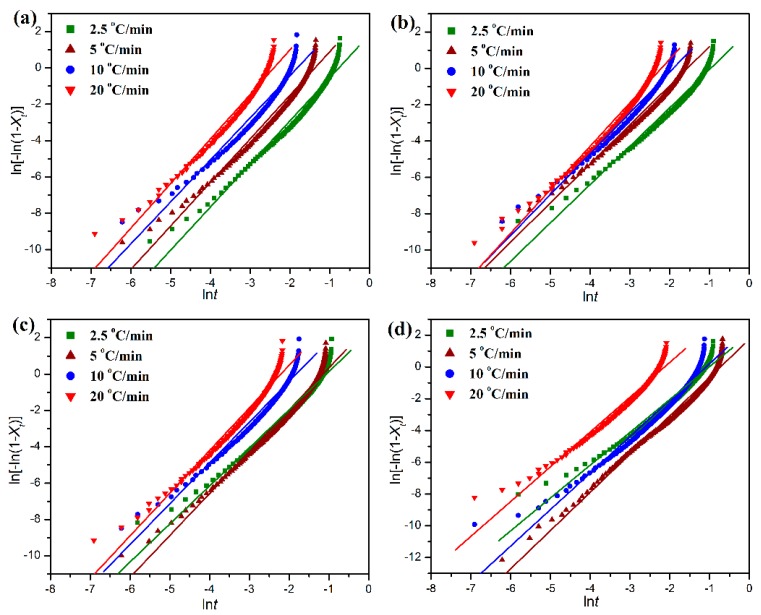
Avrami plots of ln[−ln(1 − *X_t_*)] versus ln*t* for the non-isothermal crystallization of (**a**) pure POM and its blends with (**b**) 10 wt %, (**c**) 20 wt %, and (**d**) 40 wt % of PLA at different cooling rates.

Figure 9 shows the plots of ln[−ln(1 − *X_t_*)] versus ln*t*, which gives a clear relationship between ln[−ln(1 − *X_t_*)] and ln*t* with a slope of *n* and an intercept of ln*Z*. However, since the Avrami equation is generally applied to isothermal crystallization, it is inappropriate for describing the non-isothermal crystallization process involving a constant change of temperature. Jeziorny et al. [36] suggested that the crystallization rate parameter *Z* could be corrected for the influence of cooling rate by Equation (5).

(5) $$\ln Z_{c}=\frac{\ln Z}{\phi }$$

Nevertheless, the kinetic parameter *Z_c_* does not keep a constant as suggested by Jeziorny et al. [33] at different scanning rates. The half crystallization time (*t*_1/2_) defined as the time taken to complete half of the full crystallization can be calculated by Equation (6).

(6) $$t_{1/2}={\left(\frac{\ln2}{Z}\right)}^{\frac{1}{n}}$$

Furthermore, the crystallization rate parameter (CRP) is also introduced by taking into consideration the temperature width for a crystallinity degree of 50% together with the cooling rates, and it can be determined from the slope of the plots of reciprocal *t*_1/2_ against *ϕ,* as shown in Figure S1 (see Supplementary Material) according to Equation (7) [47].

(7) $$CRP=\frac{d(1/t_{1/2})}{d(dH/dt)}$$

Furthermore, the activation energy (Δ*E_a_*) of non-isothermal crystallization can be calculated by the Kissinger’s equation as expressed by Equation (8) [48]:

(8) $$\ln\left(\frac{\phi }{T_{p}^{2}}\right)=-\frac{\Delta E_{a}}{R}\cdot \frac{1}{T_{p}}+\ln\left(\frac{A\cdot R}{\Delta E_{a}}\right)$$

where *ϕ* is the cooling rate, *T_p_* the crystallization peak temperature, *R* the gas constant, and *A* is the frequent factor. As a result, the values of Δ*E_a_* can be determined by the slope of the plot of ln(*ϕ*/*T_p_*^2^) against 1/*T_p_* as displayed in Figure S2 (see Supplementary Material). All of these kinetic parameters for the non-isothermal crystallization of pure POM and its blends with PLA are collected in Table 1. It is important to note in Table 1 that the faster the cooling rate, the shorter the half-time (*t*_1/2_) required for the completion of non-isothermal crystallization for both pure POM and its blends with PLA, indicating that the crystallization process can accelerated by improving the cooling rate. The Avrami exponent is found to range from two to three for both the pure POM and its blends with PLA at any given cooling rates, and however it presents an increasing trend with an improvement of cooling rate in most cases, suggesting an increase of dimension in the crystal growth with increasing the cooling rate. On the other hand, the *t*_1/2_ value tends to increase with the incorporation of PLA into POM, whereas the Avrami exponent seems to decrease. It is found in Table 1 that, in most cases, the *t*_1/2_ values of POM/PLA blends are improved with increasing the PLA content at the same cooling rate under the non-isothermal condition. Although the PLA domain is in the melt state when the crystallization of POM domain occurs, the high viscose PLA chains as alien species can act as nuclei to induce the heterogeneous nucleation of POM domain. However, there are some exceptions observed in the *t*_1/2_ value, indicating that the PLA domain serves a dual role as a nucleating agent and an obstacle due to entanglement, thus resulting in a complicated trend. On the other hand, the crystallization rate parameter is also found to decrease as observed in Table 1, confirming the decreasing trend on a single scale of crystallization rate for the blends. It is understandable that the crystallization temperature of PLA is far lower than that of POM. Therefore, the amorphous PLA macromolecules may disturb the non-isothermal crystallization process of POM domain in the crystallization temperature range of POM and reduces the crystallization rate of blends. Although the introduction of PLA into POM leads to a decrease of the Avrami exponent at any cooling rates, the Avrami exponents of the blends are found to tend to decrease with increasing the PLA content. This result implies that the PLA macromolecules not only can act as athermal nuclei to promote the crystal growth of POM but also can hinder the molecular motion of POM domain and reduces its crystal dimensions accordingly.

The effective energy barrier for non-isothermal crystallization process of pure POM and its blends with PLA can be determined by the values of Δ*E_a_* shown in Table 1. It is noteworthy that the values of crystallization activation energy of pure POM, PLA, and their blends are negative, indicating that all of them have an initiative crystallization capability. The value of Δ*E_a_* is found to show a decrease in the presence of 10 wt % PLA and then increases monotonically with increasing the content of PLA. This phenomenon indicates that the small amount of PLA can reduce the energy barrier of crystallization by the heterogeneous nucleation effect and promotes the crystallization of POM domain accordingly. This makes the POM domain tend to crystallize more easily and also gains a faster growth rate for crystallization. Nevertheless, it is more difficult for POM domain to crystallize at higher contents of PLA, because the large number of PLA chains may act as obstacles to the motion and diffusion of POM macromolecules, thus depressing their crystal growth in the overall non-isothermal crystallization process.

**Figure 10:**
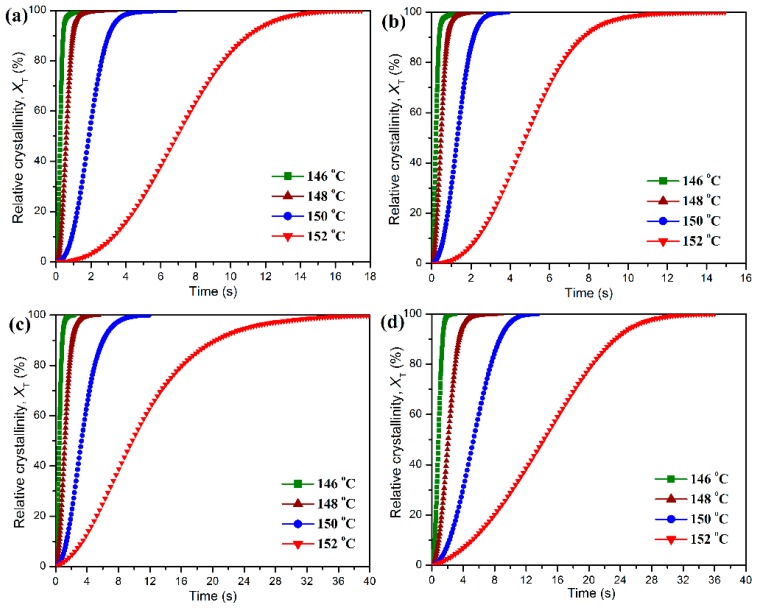
Development of relative degree of crystallinity as a function of time for the isothermal crystallization of (**a**) pure POM and its blends with (**b**) 10 wt %, (**c**) 20 wt %, and (**d**) 40 wt % of PLA at different crystallization temperatures.

The isothermal crystallization behaviors and kinetics of POM and its blends with PLA were also studied by DSC scans at different crystallization temperatures. The development of relative degree of crystallinity as a function of crystallization time can be obtained from the DSC scans under the isothermal condition by Equation (9):

(9) $$X_{t}=\frac{\int_{0}^{t}\left(\frac{dH_{c}}{dt}\right)dt}{\int_{0}^{\infty }\left(\frac{dH_{c}}{dt}\right)dt}\times 100\%$$

where *X_t_* is the relative degree of crystallinity, *dH*_c_/*dt* the heat flow rate of DSC scans and *t* is an arbitrary crystallization time. Figure 10 shows the plots of *X_t_* against *t* at different crystallization temperatures for the isothermal crystallization of pure POM and its blends with PLA. It can be seen in Figure 10 that all of these curves show a similar sigmoid shape, and the crystallization time for all samples seems to shorten with a decrease of crystallization temperature. Compared to pure POM, the PLA blend containing 10 wt % PLA exhibits a faster development in the relative degree of crystallinity at the given crystallization temperatures. However, the development of relative degree of crystallinity seems to become slower when the PLA content is further improved. These results indicate that the loading of PLA significantly influences the isothermal crystallization of POM domain in the blend. The isothermal crystallization kinetics of pure POM and its blends with PLA were studied by means of the well-known Avrami equation [37,38], which establishes the relationship between the development of *X_t_* against *t* by Equation (10):

(10) $$1-X_{t}=\exp(-Kt^{n})$$

where *K* and *n* are the Avrami crystallization rate constant and Avrami exponent, respectively. The values of *K* and *n* can be obtained from the slope of plot of ln[−ln(1 − *X_t_*)] against ln*t* by the following equation:

(11) $$\ln\left[-\ln\left(1-X_{t}\right)\right]=n\ln t+\ln K$$

**Figure 11:**
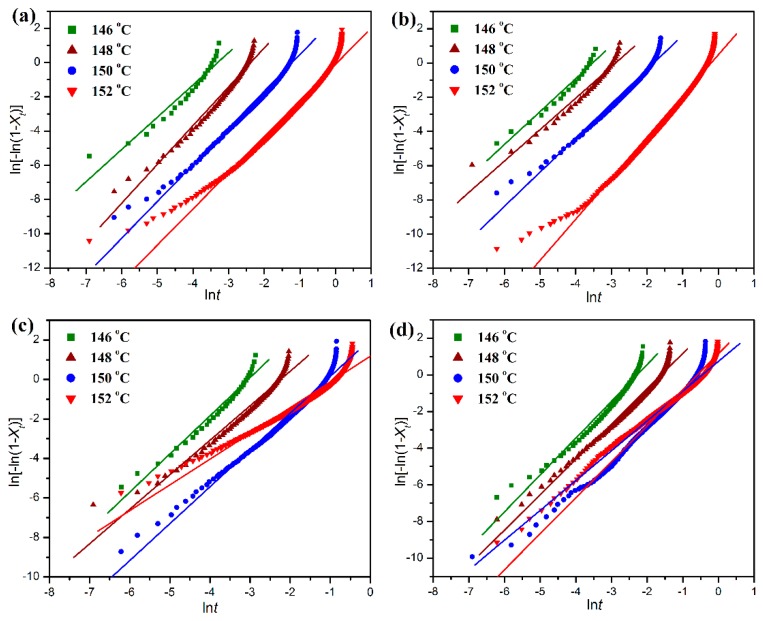
Avrami plots of ln[−ln(1 − *X_t_*)] versus ln*t* for the isothermal crystallization of (**a**) pure POM and its blends with (**b**) 10 wt %, (**c**) 20 wt %, and (**d**) 40 wt % of PLA at different crystallization temperatures.

Figure 11 shows the linear fitting plots of ln[−ln(1 − *X_t_*)] against ln*t* for the isothermal crystallization of pure POM and its blends with PLA. The half time (*t*_1/2_) of isothermal crystallization, defined as the time required for reaching an *X_t_* value of 50%, is commonly used to evaluate the crystallization rates of POM/PLA blends within different environments, and it can be calculated by Equation (12).

(12) $$t_{1/2}={\left(\frac{\ln2}{K}\right)}^{\frac{1}{n}}$$

Moreover, as the isothermal crystallization process is assumed to be thermally activated, the crystallization rate parameter *K* can be approximately described by an Arrhenius equation as follows [49]:

(13) $$K^{1/n}=K_{0}\exp(-\Delta E_{a}/RT)$$

where *K* is a temperature-independent pre-exponential factor, Δ*E_a_* is the total activation energy consisting of the transport activation energy ΔE* and the nucleation activation energy Δ*F*. * in ΔE* refers to the activation energy required to transport molecular segments across the phase boundary to the crystallization surface. Δ*F* is the free energy of formation of the critical size crystal nuclei at crystallization temperature *T_c_*, *R* the universal gas constant and *T* is the absolute temperature. The values of Δ*E_a_* can be determined by the slope of the Arrhenius plots of ln*K*/*n* against l/*T*_c_ as shown in Figure S3 (see Supplementary Material) using the following equation:

(14) $$\frac{\ln K}{n}=\ln K_{0}-\frac{\Delta E_{a}}{RT}$$

All of these isothermal crystallization kinetic parameters derived from the analysis of the Avrami model are also summarized in Table 1. It is noted in Table 1 that for pure POM and its blends with PLA the *t*_1/2_ values all present an almost exponential increase as the crystallization temperature is improved. At the same crystallization temperature, the addition of small amounts of PLA leads to a decrease of the *t*_1/2_ value as an indicator of improved crystallization rate. This is the evidence that the crystallization of POM domain occurs by a nucleation-controlled mechanism [49]. However, the *t*_1/2_ values of the blends tend to increase continually with further increasing the PLA content, suggesting that the crystallization of POM domain has been controlled by the molecular-confined mechanism [49]. It is apparent that the serious entanglement between POM and PLA chains takes place in the melt state at a high POM loading. Accordingly, the motion of POM chains is hindered during the isothermal crystallization process, resulting in a decrease of crystallization rate.

It is also clearly observed in Table 1 that the Avrami exponents of pure POM are in the range of 1.89–2.27, implying athermal nucleation and three-dimensional growth. However, the Avrami exponent tends to decrease in the presence of PLA in most cases, suggesting athermal nucleation and two-dimensional growth for the POM domain due to the confinement of molecular motion. These results are in good agreement with the non-isothermal crystallization data. It is necessary to discuss the effect of PLA on the activation energy (Δ*E_a_*) of isothermal crystallization behaviors of POM domain in the blends. As seen in Table 1, the Δ*E_a_* value of the blend decreases slightly in the presence of 10 wt % PLA, and furthermore, the Δ*E_a_* values show a continual increase as the PLA content is further improved. Such a trend is in perfect agreement with the non-isothermal crystallization results. Normally, there are two factors determining the crystallization rate of a polymer when another polymer is introduced. On the one hand, the additive polymer can play a role of heterogeneous nucleation in the crystallization process of the host polymer in the blend thus generating a positive effect on crystallization. On the other hand, the additive polymer can hinder the motion and diffusion of host polymer chains to the surface of nucleus by chain entanglement, and therefore it constrains the crystalline growth by a confinement mechanism in the blend, which leads to a negative effect on crystallization. The non-isothermal and isothermal crystallization kinetic results confirm that the PLA domain at a low content can serve as a nucleating agent to reduce the energy barriers for the non-isothermal and isothermal crystallization of POM domain. However, at the high content of PLA, the polymeric chains from POM and PLA domains are highly entangled into each other in the melt state. It is well known that the polymer chains must overcome certain energy barriers to diffuse and attach onto the growing surface of a crystal, and therefore the presence of large amounts of PLA chains may confine the motion of POM chains and hinder their crystal growth by imposing the entanglement with the POM chains. As a result, the Δ*E_a_* value is improved and the crystallization rate is reduced.

**Figure 12:**
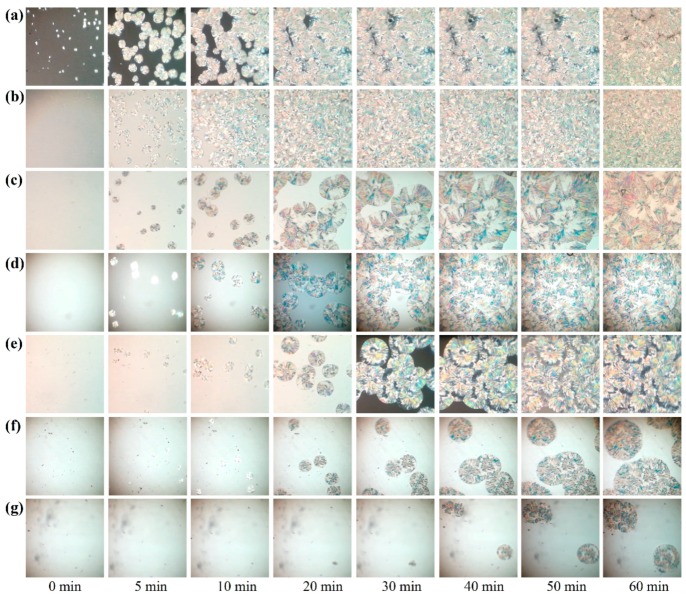
Polarizing optical microscopic images of (**a**) pure POM and its blends with (**b**) 10 wt %, (**c**) 20 wt %, (**d**) 30 wt %, (**e**) 40 wt %, (**f**) 50 wt %, and (**g**) 60 wt % of PLA during the isothermal crystallization process.

The real isothermal crystallization processes for pure POM and its blends with PLA were monitored by a polarizing optical microscope, and the obtained microscopic images are shown in Figure 12. It is clearly observed that pure POM performs a fast crystallization process at a given temperature of 145 °C and forms a number of spherulites within 60 min. Compared to pure POM, the blend containing 10% PLA is found to present a slightly slower crystallization process during the same period and show a much smaller size of spherulites due to the nucleating effect of PLA. The number of spherulites is found to increase. However, the crystallization process becomes much slower with a further increase of PLA content. On the other hand, the spherulite size of the blends at the PLA content over 10 wt % become larger than that with 10 wt % of PLA, indicating that the confinement dominates the nucleation and crystallization of POM domain at the high loadings of PLA. These results confirm the conclusions obtained from the isothermal crystallization kinetic study.

### 3.5. Thermal Degradation Kinetics and Lifespan Prediction

**Figure 13:**
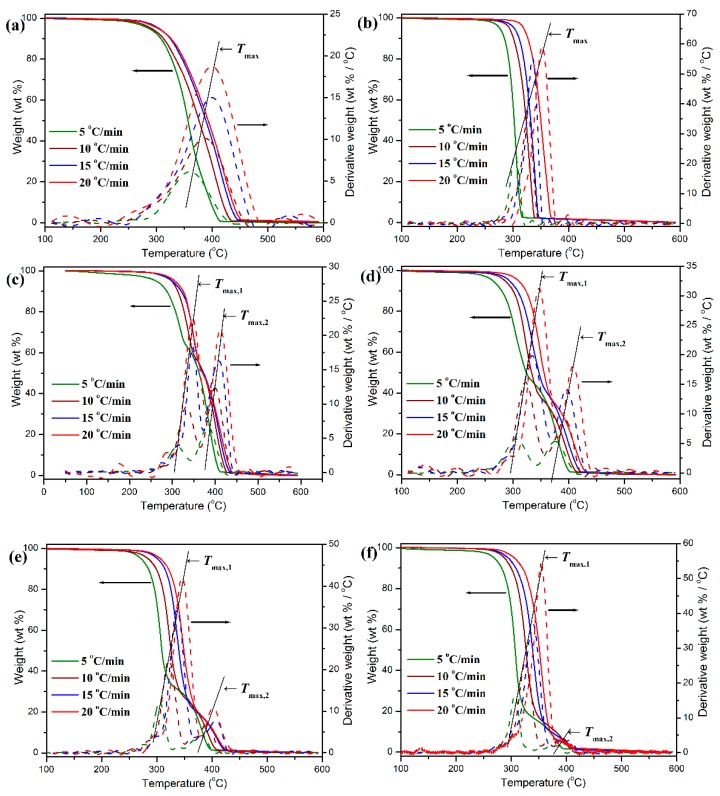
TGA and derivative thermogravimetric (DTG) thermograms of (**a**) pure POM, (**b**) pure PLA, and its blends with (**c**) 10 wt %, (**d**) 20 wt %, (**e**) 30 wt %, (**f**) 50 wt % of PLA during the isothermal crystallization process.

TGA is considered as a powerful tool to study the thermal degradation kinetics of polymers. TGA methodology can provide important information the degradation activation energy (Δ*E_a_*), reaction order (*n*), and frequency factor (*A*) in terms of various kinetic models, and consequently, the lifetime of polymers at various temperatures can be predicted on the basis of these kinetic parameters obtained from the relevant kinetic models [39,40,50,51,52,53]. The thermal degradation behavior and degradation kinetics of pure POM, PLA, and their blends were evaluated by TGA at different heating rates, and some representative TGA thermograms are illustrated in Figure 13. The degradation kinetic data including the initial degradation temperature (*T_i_*) corresponding to 5 wt % weight loss and final degradation temperature (*T_f_*) corresponding to 5 wt % residual char left calculated at different heating rates for pure POM, PLA, and POM/PLA blends are collected in Table 2. It is clearly observed in Figure 13 that both pure POM and PLA exhibit a typical one-step degradation behavior at the given heat rates in the temperature ranges of 250–450 and 250–360 °C, respectively. It is interesting to note that the TGA thermograms for these two polymers shift toward a higher temperature with improving the heat rate. This result suggests that the polymer has to complete thermal decomposition in a higher temperature region due to slow heat diffusion in the faster heating process [53]. Oppositely, in the slower heating process, i.e., at a low heating rate, the polymer can rapidly achieve an equilibrium in thermal decomposition and thus complete the thermal degradation in a lower temperature region. The characteristic temperature at the maximum weight-loss rate (*T*_max_) can be adopted as an important index to indicate the thermal stability of a polymer and obtained from the DTG thermogram. It is found in Figure 13 and Table 2 that the *T*_max_’s of pure POM and PLA were distributed in the temperature range of 359.2–399.4 and 304.2–353.6 °C at the given four heating rates, respectively, indicating that pure POM has much better thermal stability than pure PLA. There are two *T*_max_’s observed in the DTG thermograms of POM/PLA blends, in which the lower characteristic temperature (*T*_max,1_) is attributed to PLA domain and the higher characteristic one (*T*_max,2_) is associated with the thermal degradation of POM domain. It is noteworthy that the incorporation of 10 wt % PLA results in a considerable improvement in the *T*_max_’s of POM and PLA domains in the blend. This phenomenon may be ascribed to the enhancement of crystallinity for POM domain as well as the envelopment effect of PLA domain in the blends. It is accepted that the high crystallinity improves the heat resistance of POM domain, while the envelopment with POM domain effectively prevents the heat diffusion to PLA domain. However, the increment becomes less with further increasing the PLA content. As seen in the DTG thermograms of Figure 13, both *T*_max__,1_ and *T*_max__,2_ tend to decrease with reducing the heating rate, indicating that both POM and PLA domains exhibit a poorer thermal stability on a longer time scale due to the longer heating time caused by a lower heating rate.

The thermal degradation kinetics of pure POM, PLA, and their blends were studied by means of the Kissinger’s and Flynn–Wall’s methods in this work. For the thermal decomposition reaction of a polymer, the conversion (*a*) of decomposition reaction is defined as the ratio of mass loss at arbitrary time to total mass loss at complete decomposition temperature from TGA analysis and can be calculated by the following equation:

(15) $$\alpha =\frac{w_{0}-w_{t}}{w_{0}-w_{f}}$$

where *w*_0_, *w*_t_, and *w*_f_ are the initial weight, actual weight at time *t*, and, final weight of the sample at the end of degradation, respectively. The thermal degradation kinetics of this polymer can be expressed by Equation (16) on the basis of the reaction rate for a basic solid-state chemical reaction [54]:

(16) $$d\alpha /dT=k{\left(1-\alpha \right)}^{n}$$

where *α* is the conversion of thermal decomposition, *k* the rate constant, and *n* is the apparent order of reaction. The combination of the above two expressions with the Arrhenius expression gives the following relationship, which is the basis of numerous analytical approaches to the calculation of kinetic parameters from TGA data [Equations (17) and (18)]:

(17) $$k=A\exp(-\Delta E_{a}/RT)$$

(18) $$d\alpha /dT=A{\left(1-\alpha \right)}^{n}\exp(-\Delta E_{a}/RT)$$

The degradation temperatures of pure POM, PLA, and their blends at different conversion levels are summarized in Table S1 (see Supplementary Material). These data indicate that the incorporation of small amounts of PLA seems not to influence the evolution of degradation reaction of the blends, and however the high content of PLA leads to a significant decrease of degradation temperature for the blends at the same conversion. According to Kissinger’ method [51], the peak temperatures given by the maxima of the first derivative weight-loss thermogram can be used to calculate the apparent activation energy (Δ*E_a_*) of thermal degradation reaction by means of the following expression:

(19) $$\ln\left(\frac{\beta }{T_{\max}^{2}}\right)=\ln\left(\frac{A\cdot R}{\Delta E_{a}}\right)-\frac{\Delta E_{a}}{R}\cdot \frac{1}{T}$$

where *β* is the heating rate, *T* the absolute temperature corresponding to the conversion, and *R* is the gas constant. On the other hand, a new expression was developed by Flynn and Wall to calculate the thermal degradation kinetic parameters of a polymer based on the Kissinger’s equation as follows [39]:

(20) $$\ln\beta =\ln\left(\frac{Z\cdot \Delta E_{a}}{R}\right)-\ln\alpha -0.4567\frac{\Delta E_{a}}{R}\cdot \frac{1}{T}$$

According to the Flynn–Wall’s method, the apparent activation energy of thermal degradation reaction for pure POM, PLA, and their blends could be obtained from the slope of the linear plot of ln*β* versus 1/*T* at a fixed conversion (normally taken as 5% conversion) [39]. Figure S4 shows the Flynn–Wall plots of ln*β* versus 1/*T* at the 5% conversion for all the samples (see Supplementary Material), and Table 2 gives the apparent activation energy of thermal degradation derived from the Flynn–Wall plots. It is calculated that the apparent activation energy for the thermal degradation reactions of pure POM and PLA at a conversion of 5% is 371.8 and 309.2 kJ/mol, respectively, indicating that PLA performs thermal decomposition earlier than POM. The apparent activation energy shows a remarkable decrease with the addition of 5 wt % PLA into POM and reaches a minimum with the PLA content rising up to 20 wt %. The apparent activation energy of POM/PLA blends tends to increase continuously with a further increase of PLA content. It is well known that the apparent activation energy is associated with the initial decomposition stage [54]. The degradation kinetic results explicitly suggest that the incorporation of small amounts of PLA can initiate the thermal degradation reaction more easily.

**Figure 14:**
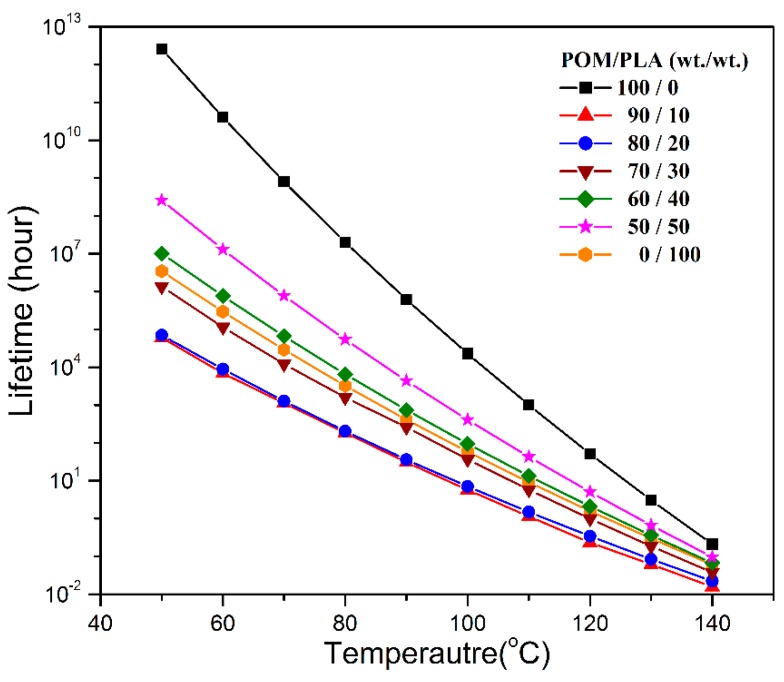
Plots of lifespan as a function of temperature predicted by the Loop method for pure POM, PLA, and their blends.

The lifespans of pure POM, PLA, and their blends in the temperature range of 50–140 °C were further predicted according to the method proposed by Toop et al. [40] using Equations (21)–(23):

(21) $$X_{f}=\Delta E_{a}/RT_{5\%}$$

(22) $$\ln p(X_{f})=-2.315-0.457X_{f}$$

(23) $$\ln t_{f}=\frac{\Delta E_{a}}{RT_{f}}+\ln\left(\frac{\Delta E_{a}}{\beta \cdot R}\cdot p(X_{f})\right)$$

where *X_f_* is set as Δ*E_a_*/*RT*_5%_, *T*_5%_ the absolute temperature at 5% weight loss, *T_f_* the failure temperature, *t_f_* the approximate time of failure, and ln *p*(*X_f_*) is the linear function of *X_f_*. The lifespans of pure POM, PLA, and their blends at different temperatures was calculated by Equation (23) on the basis of the Δ*E_a_* values obtained from the Flynn–Wall method, and the results of lifespan prediction are presented in Figure 14. It is clearly observed that pure POM exhibits a much longer lifespan than pure PLA in the temperature range between 50 and 140 °C due to its structural stability. Moreover, the temperature is also found to generate a significant effect on the lifespans of these two polymers. The higher the temperature, the shorter is the lifespan as a result of the effect of thermal stability. The lifespans of POM/PLA blends are found to decrease significantly in the presence of 10 wt % PLA at all temperatures and then tends to increase with an increase of PLA content. It is noted that the lifespans of POM/PLA blends are shorter than pure PLA as long as the content of PLA is lower than 30 wt %. On the basis of the Flynn–Wall’s method and Loop’s model, the blends at the PLA contents of 10%, 20% and 30% were predicted to have the lifespans of about 6.0 × 10^4^, 7.03 × 10^4^, and 1.37 × 10^6^ h at 50 °C, respectively. However, the lifespan of pure POM is predicted to be 2.56 × 10^1^^2^ h at 50 °C. Evidently, the introduction of 10 wt % PLA into POM can lead to the shortest lifespan in all of the POM/PLA blending samples. This percentage of PLA favors the natural degradation of the blend as well as the reservation of better mechanical performance within the lifespan of the blends.

### 3.6. Enzymatic Degradation Behavior

**Figure 15:**
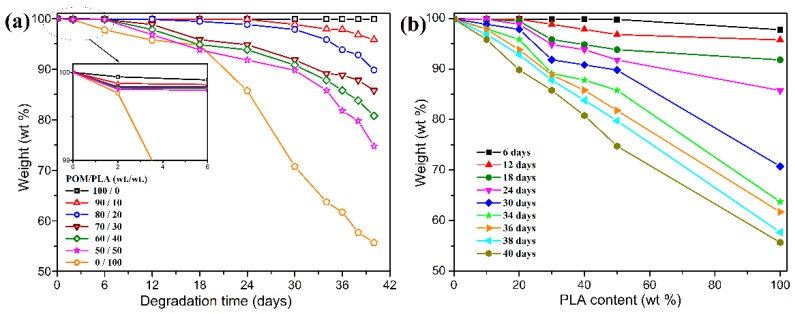
(**a**) Plots of mass loss as a function of degradation time and (**b**) plots of mass loss as a function of PLA content for POM/PLA blends.

PLA has been confirmed to be a full biodegradable thermoplastic polymer [3]. In order to determine the partial biodegradability of POM/PLA blends, their enzymatic degradation behaviors were investigated for pure POM, PLA, and their blends. Figure 15 shows the mass loss as a function of degradation time for these pure resins and their blends. As expected, pure PLA exhibits a weight loss of about 45 wt % in comparison of its original weight within 40 days due to an enzymatic degradation effect. However, pure POM only exhibits a smallest mass loss of approximately 0.2 wt % within the same days, which means that POM is non-biodegradable due to its high degree of crystallinity and stable molecular structure [55,56]. It is noteworthy that the mass loss of the blends increases gradually with an increase of PLA content and reaches up to 25.3% when the PLA content is 50 wt %, indicating that the POM/PLA blends are partially biodegradable. The degradation activity of the blends is derived from the PLA phase, and the enzymatic fluid may migrate into the inside of the blends and contacts the PLA phase, thus resulting in its biodegradation. It is also observed in Figure 15 that the blends are enzymatically degraded rapidly after 24 days. The initial biodegradation within 24 days can enlarge the molecular gaps of the blends and enhances the penetrating power of enzymatic fluid. As a result, the degradation rate of the blends is improved significantly at the later enzymatic degradation stage. Moreover, the higher content of PLA is found to lead to a faster degradation rate at the later degradation stage due to the presence of larger molecular gaps in the blends.

**Figure 16:**
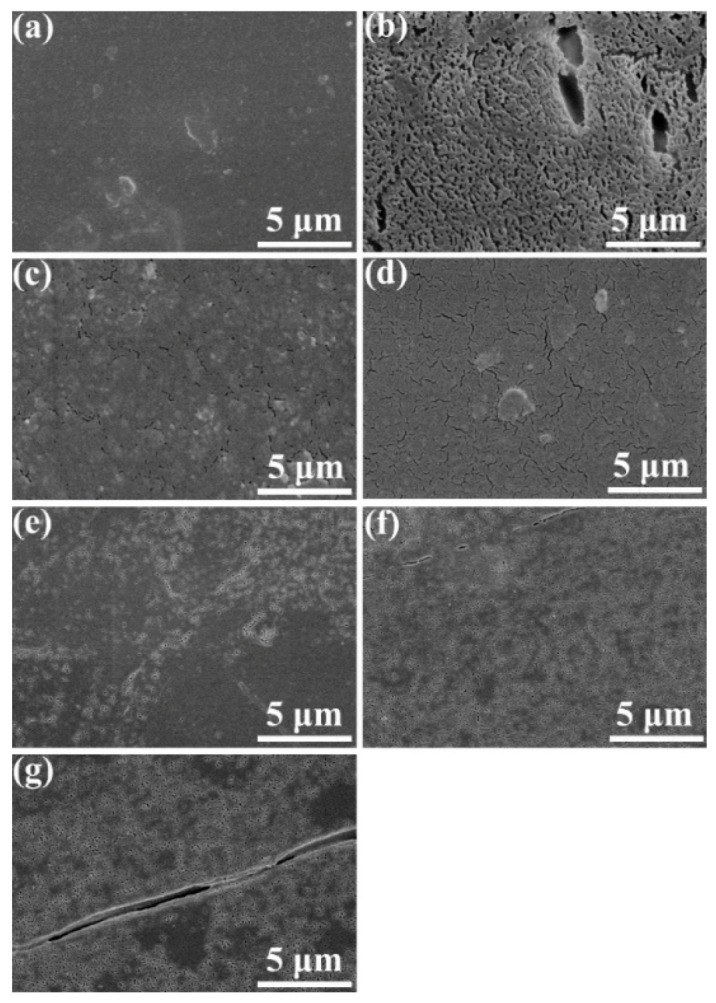
SEM micrographs of (**a**) pure POM, (**b**) pure PLA, and their blends with (**c**) 10 wt %, (**d**) 20 wt %, (**e**) 30 wt %, (**f**) 40 wt % and (**g**) 50 wt % PLA after enzymatic degradation.

The surface morphology of specimens after enzymatic degradation was observed with SEM and the resulting SEM micrographs are illustrated in Figure 16. Pure POM is observed to show a smooth surface without etching trace, whereas there are a large number of holes found on the surface of pure PLA after enzymatic degradation over 40 days. On the other hand, a few cracks can be observed on the surface of the blend containing 10 wt % PLA. With an increase of PLA content, the number and size of cracks is found to become larger due to the enhancement in migration of enzymatic fluid and enzymatic degradation effect. When the PLA content increases up to 30 wt %, some holes appear on the surface of specimens instead of cracks. The number and size of these holes also become larger with the further improvement of PLA content. These phenomena confirm that the POM/PLA blends are partially biodegradable, and the biodegradable level can be improved by increasing the loading PLA in the blends. Such partial biodegradability can lead to the formation of porous microstructure in the blends after enzymatic degradation and further results in the collapse of bulk blends, which are advantageous to the garbage disposal of plastic wastes. Considering the acceptable mechanical performance in tensile, flexural, and impact strength and the crystallization performance for processability as well as partial biodegradability, it will be a reasonable selection to keep a low loading of PLA in the range of 10–20 wt % so as to maintain the optimum comprehensive performance for the POM/PLA blending system.

## 4. Conclusions

The POM/PLA blends were developed as a type of potentially biodegradable material by a melt-extrusion processing technology, and their mechanical properties, crystallization behavior and kinetics, thermal degradation kinetics and stability, lifespan prediction and enzymatic degradation behavior were investigated extensively. The POM and PLA domains were confirmed to be partially miscible in the melt state at low temperature and become phase-separated at elevated temperatures, leading to a typical lower-critical-solution-temperature behavior. The blends also presented two distinct glass transition temperatures (*T_g_*) at any mass ratios when quenched from the homogeneous state, and both POM and PLA domains showed an apparent depression in their respective *T_g_*’s in the blends. Owing to the partial miscibility between two domains, the tensile strength and impact toughness of POM/PLA blends gradually decreased with an increase of PLA content, but their flexural strength and modulus presented an increasing trend with PLA content. Based on the non-isothermal and isothermal crystallization kinetic studies, the crystallization of POM domain in the blends was controlled by the molecular-confined mechanism, and the serious entanglement between POM and PLA chains occurring in the melt state hindered the motion of POM domain, which resulted in a continual decrease in crystallization rates of the blends with increasing the PLA content. The introduction of PLA into POM not only led to a slight increase of thermal stability of POM domain at low PLA contents but also shortened the lifespan of the blends, favoring the natural degradation of the blend. The POM/PLA blends exhibited an improvement in biodegradable level with an increase of PLA content, and their mass loss reached up to 25.3 wt % at the end of 48-h enzymatic degradation when 50 wt % of PLA was incorporated.

## Figures and Tables

**Table 1:** The crystallization kinetic parameters of pure POM and its blends with PLA under the non-isothermal and isothermal conditions.

POM/PLA Blend (wt/wt)	Non-Isothermal Crystallization							Isothermal Crystallization				
	*ϕ* (°C/min)	*T_p_* (°C)	Z*_c_* (1/min^n^)	*N*	*t*_1/2_ (min)	CRP (K^−1^)	Δ*E_a_* (kJ/mol)	*T_c_* (°C)	*K* (1/min^n^)	*n*	*t*_1/2_ (min)	Δ*E_a_* (kJ/mol)
100/0	2.5	148.5	1.36	2.37	1.44	0.15	−446.37	146	10.29	1.89	0.24	−800.39
	5	146.8	1.49	2.40	0.81			148	2.30	2.27	0.59	
	10	144.6	1.28	2.32	0.49			150	0.18	2.15	1.89	
	20	141.7	1.23	2.46	0.30			152	1.15 × 10^−2^	2.12	6.92	
90/10	2.5	147.4	1.47	2.11	1.45	0.14	−511.98	146	12.12	1.89	0.22	−828.88
	5	146.2	1.52	2.15	0.83			148	2.68	1.84	0.48	
	10	144.3	1.32	2.25	0.51			150	0.28	2.00	1.31	
	20	141.6	1.20	2.39	0.32			152	1.63 × 10^−2^	2.40	4.77	
80/20	2.5	146.7	1.55	2.10	1.71	0.12	−358.13	146	2.17	1.93	0.47	−732.94
	5	144.2	1.38	2.31	1.14			148	5.22 × 10^−1^	1.75	1.18	
	10	141.5	1.29	2.25	0.61			150	7.43 × 10^−2^	1.87	3.30	
	20	138.2	1.19	2.35	0.43			152	3.50 × 10^−2^	1.31	9.76	
70/30	2.5	144.6	1.67	2.15	1.69	0.11	−331.45	146	1.41	2.17	0.72	−709.21
	5	141.4	1.55	2.17	0.98			148	1.46 × 10^−1^	2.19	2.04	
	10	138.4	1.45	2.30	0.69			150	3.10 × 10^−2^	1.97	4.84	
	20	135.5	1.11	2.41	0.38			152	9.68 × 10^−3^	1.69	12.52	
60/40	2.5	144.1	1.50	2.06	1.82	0.11	−297.16	146	9.60 × 10^−1^	2.01	0.85	−670.59
	5	141.2	1.34	2.41	1.48			148	1.21 × 10^−1^	1.93	2.08	
	10	138.3	1.41	2.29	0.87			150	2.63 × 10^−2^	1.96	5.31	
	20	135.0	1.05	2.19	0.40			152	6.12 × 10^−3^	1.83	13.25	
50/50	2.5	142.4	2.63	2.11	1.72	0.09	−291.40	146	1.43 × 10^−1^	2.31	1.99	−582.79
	5	139.5	1.61	2.20	1.09			148	3.79 × 10^−2^	2.37	3.41	
	10	136.2	1.41	2.18	0.75			150	3.10 × 10^−2^	1.62	6.81	
	20	132.2	1.08	2.32	0.46			152	5.78 × 10^−3^	1.60	19.92	

**Table 2:** The thermal degradation kinetic date obtained from thermogravimetric (TGA) measurements for pure POM, PLA, and their blends.

POM/PLA Blend (wt/wt)	*β* (°C/min)	*T*_onset_ (°C)	*T*_max,1_ (°C)	*α*_max,1_ (%/min)	*T*_max,2_ (°C)	*α*_max,2_ (%/min)	*T*_end_ (°C)	Δ*E_a_* (kJ/mol)
100/0	5	285.6	–	–	360.1	6.1	402.6	371.8
	10	290.1	–	–	386.8	10.1	422.7	
	15	304.2	–	–	398.5	15.0	434.7	
	20	301.9	–	–	399.1	18.7	440.9	
90/10	5	260.6	311.6	3.7	382.8	6.6	399.5	184.2
	10	298.6	332.9	9.6	399.4	12.3	419.4	
	15	299.4	347.1	18.3	409.0	16.3	425.3	
	20	305.5	347.0	22.5	412.1	20.9	428.9	
80/20	5	263.2	305.9	5.7	376.0	5.3	391.6	182.6
	10	285.2	322.8	15.2	393.4	9.0	402.6	
	15	292.8	334.5	19.7	396.4	14.1	410.2	
	20	309.6	346.9	31.4	408.1	18.0	420.0	
70/30	5	270.8	305.0	12.4	382.5	4.0	388.1	220.3
	10	283.5	322.0	22.2	391.4	7.0	396.0	
	15	300.9	336.8	34.2	402.3	7.8	410.2	
	20	305.9	345.7	41.9	407.2	10.9	411.3	
60/40	5	268.5	306.6	16.3	383.2	4.1	385.7	232.7
	10	287.7	326.1	26.0	392.7	6.9	393.3	
	15	294.5	339.2	37.1	395.5	7.4	397.5	
	20	305.3	355.1	54.2	399.4	11.3	401.9	
50/50	5	273.5	308.9	16.5	359.2	2.7	363.4	267.9
	10	282.7	327.2	26.2	361.6	5.8	368.6	
	15	297.3	341.3	35.2	367.4	6.9	372.8	
	20	301.7	352.7	34.5	369.8	9.5	373.8	
0/100	5	276.1	303.7	22.7	–	–	313.4	309.2
	10	293.8	326.9	35.8	–	–	336.9	
	15	302.4	335.3	55.3	–	–	344.6	
	20	319.3	352.5	59.1	–	–	366.2	

## Supplementary Materials

The following are available online at https://www.mdpi.com/2073-4360/11/9/1516/s1, Figure S1: Plots of reciprocal half-time (1/*t*_1/2_) of crystallization as a function of cooling rate for pure POM and its blends with PLA, Figure S2: *Kissinger* plots of ln(*φ*/*T*_p_^2^) versus 1/*T*_p_ for the non-isothermal crystallization of pure POM and its blends with PLA, Fiture S3: *Arrhenius* plots of lnK/n versus l/Tc for the isothermal crystallization of pure POM and its blends with PLA, Figure S4: Flynn–Wall plots of ln*β* versus l/*T*_max_ for the thermal degradation reaction of POM/PLA blends, Table S1: The thermal degradation temperatures as a function of percentage conversion for pure POM, PLA and POM/PLA blends at different heating rates.
