# Supplementary material for: Development of Polyoxymethylene/Polylactide Blends for a Potentially Biodegradable Material: Crystallization Kinetics, Lifespan Prediction, and Enzymatic Degradation Behavior
Source: Polymers (Basel). 2019 Sep 18;11(9):1516. doi: 10.3390/polym11091516 (PMC6780351; doi:10.3390/polym11091516)
Supplement: Supplementary file 1 [file polymers-11-01516-s001.pdf]

Supplementary Materials

Development of Polyoxymethylene/Polylactide Blends for a Potentially Biodegradable Material: Crystallization Kinetics, Lifespan Prediction and Enzymatic Degradation Behavior

Jianhua Li [1,2], Yatao Wang [2], Xiaodong Wang [1,*] and Dezhen Wu [1,*]

[1] State Key Laboratory of Organic–Inorganic Composites, Beijing University of Chemical Technology, Beijing 100029, China

[2] Coal Chemical R & D Center, Kailuan Group Limited Liability Corporation, Tangshan, Hebei 063018, China

* Correspondence: wangxdfox@aliyun.com (X.W.); wdz@mail.buct.edu.cn (D.W.); Tel.: +86-10-6442-1693 (X.W.)

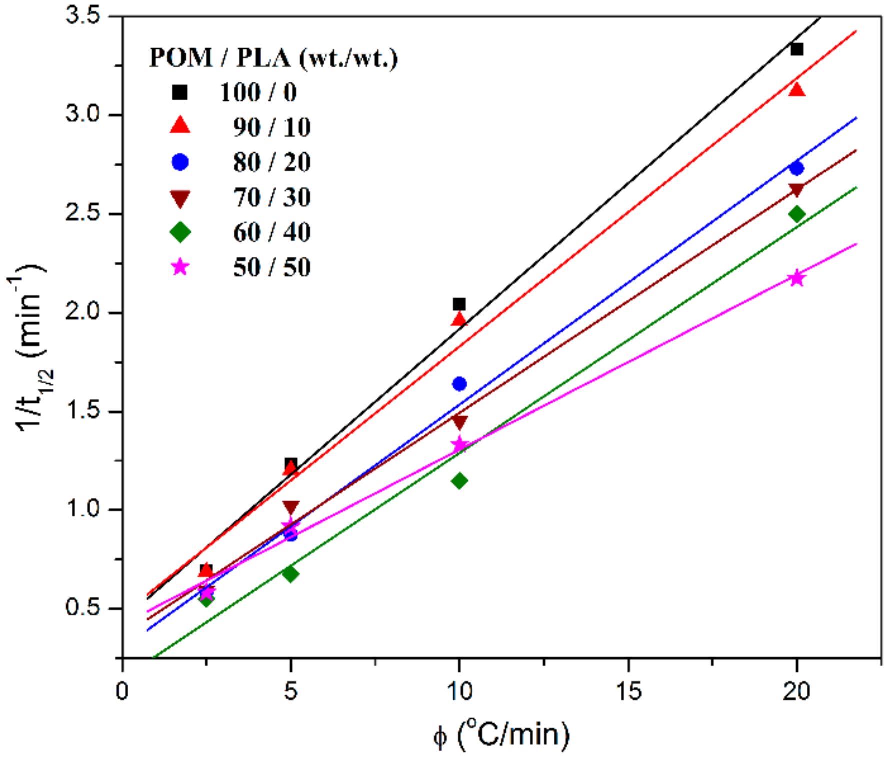

Figure S1 Plots of reciprocal half-time ($1/t_{1/2}$) of crystallization as a function of cooling rate for pure POM and its blends with PLA.

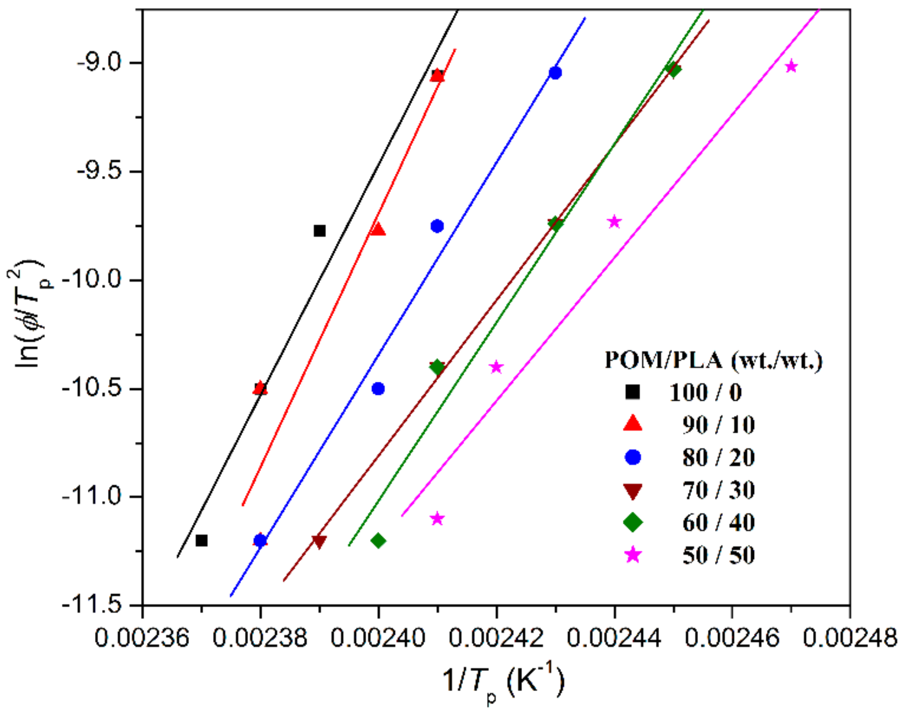

Figure S2 *Kissinger* plots of ln(ϕ/T_P^2) versus 1/T_P for the non-isothermal crystallization of pure POM and its blends with PLA.

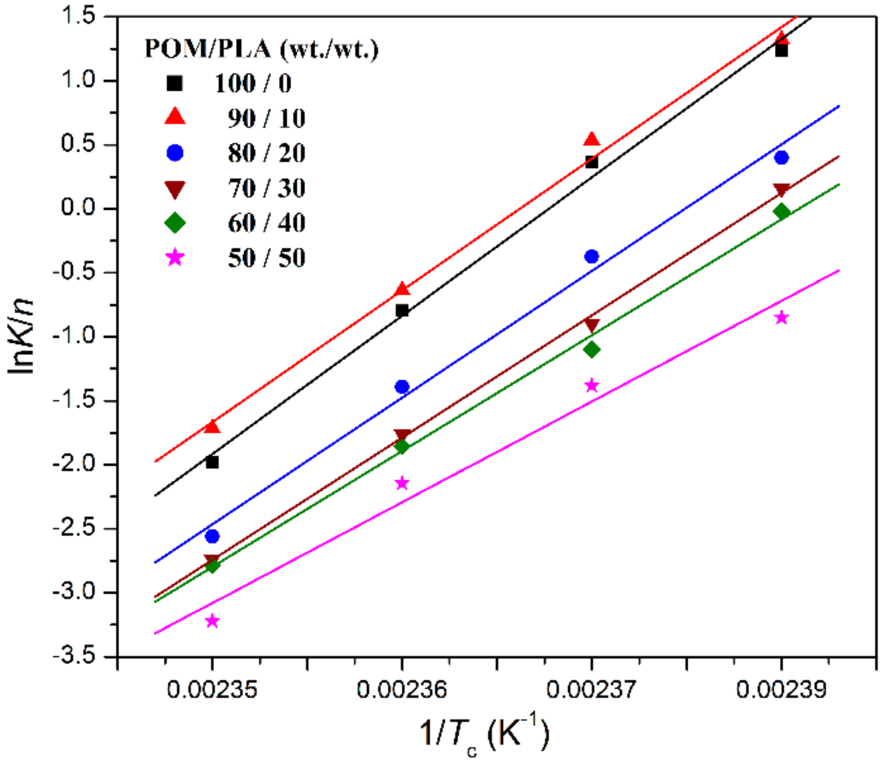

Figure S3 *Arrhenius* plots of lnK/n versus l/Tc for the isothermal crystallization of pure POM and its blends with PLA.

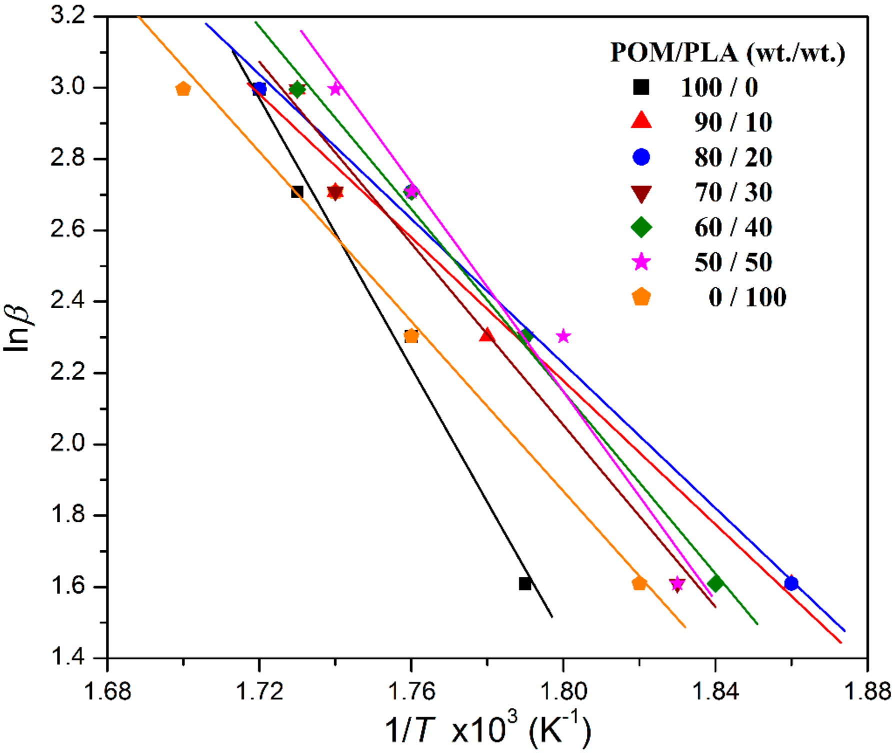

Figure S4 Flynn-Wall plots of lnβ versus l/T_{max} for the thermal degradation reaction of POM/PLA blends.

Table S1. The thermal degradation temperatures as a function of percentage conversion for pure POM, PLA and POM/PLA blends at different heating rates.

β (°C/min)	Conversion (%)	Thermal degradation temperature (°C)						
		Pure POM	Pure PLA	POM+10 wt % PLA	POM+20 wt % PLA	POM+30 wt % PLA	POM+40 wt % PLA	POM+50 wt % PLA
5	5	285.6	277.1	275.0	265.1	272.0	270.4	273.6
	10	307.5	285.3	286.8	279.6	284.6	283.1	284.1
	20	326.7	291.8	306.6	294.7	295.8	293.9	294.0
	30	339.3	296.3	318.8	303.5	301.6	300.1	299.8
	40	348.5	299.6	338.6	312.0	305.7	303.5	304.0
	50	355.9	301.9	359.7	322.6	308.9	306.8	307.4
	60	363.0	304.3	370.8	348.7	314.9	309.5	310.4
	70	371.5	306.4	379.3	365.6	339.6	313.2	313.5
	80	382.9	309.0	386.8	376.8	368.2	321.6	317.3
	90	395.3	311.4	394.8	386.0	381.7	360.7	329.3
10	5	296.6	293.7	290.9	284.6	288.8	287.7	287.8
	10	313.7	300.9	312.6	299.7	299.0	299.6	293.5
	20	336.1	309.7	327.3	312.4	310.9	312.2	305.7
	30	350.9	315.7	337.3	319.6	316.8	318.6	313.8
	40	363.8	319.9	347.7	325.3	321.1	322.6	319.7
	50	375.2	323.8	371.9	332.7	325.2	326.8	324.1
	60	386.7	326.2	385.8	346.8	331.1	330.1	328.2
	70	397.1	329.2	395.6	367.7	339.6	334.6	331.8
	80	406.7	332.1	403.8	384.6	369.7	342.8	336.0

	90	416.6	335.1	413.3	396.8	388.9	376.6	344.6
15	5	304.2	302.4	300.1	293.8	300.9	295.6	297.3
	10	322.3	311.0	320.3	307.5	314.4	308.4	306.4
	20	345.6	319.1	338.1	321.6	325.6	321.8	318.1
	30	361.6	324.2	347.6	330.6	331.1	329.2	325.7
	40	375.1	328.8	356.0	337.6	335.7	334.4	331.7
	50	387.7	331.8	372.0	345.7	339.5	338.5	336.7
	60	399.0	334.8	389.0	358.8	344.6	342.8	341.3
	70	409.1	337.6	400.1	378.9	350.9	346.5	345.6
	80	418.6	340.1	409.6	392.6	368.0	352.6	350.5
	90	428.6	342.8	418.8	403.7	400.7	369.2	363.2
20	5	307.3	318.5	305.5	309.4	306.2	305.3	302.0
	10	325.6	326.6	324.5	322.8	320.5	320.2	312.9
	20	348.9	335.6	338.9	335.5	332.9	333.3	326.4
	30	364.8	341.7	347.8	342.6	339.6	341.1	334.8
	40	378.8	346.1	355.7	348.6	344.6	346.6	340.9
	50	391.5	349.8	368.5	354.6	348.7	350.7	346.5
	60	403.4	353.6	388.1	363.4	353.5	354.6	350.9
	70	414.2	356.8	401.6	384.9	359.7	358.1	355.7
	80	423.7	360.6	412.4	401.8	371.5	363.0	360.0
	90	434.5	364.5	422.9	414.1	401.0	369.1	366.0
